# Plant Photodynamic Stress: What's New?

**DOI:** 10.3389/fpls.2018.00681

**Published:** 2018-05-23

**Authors:** Mohammad Issawi, Vincent Sol, Catherine Riou

**Affiliations:** Laboratoire Peirene (EA7500), Faculté des Sciences et Techniques, Université de Limoges, Limoges, France

**Keywords:** 5-aminolevulinic acid, diphenyl ether herbicides, photosensitizers, plant photodynamic stress, porphyrins, tetrapyrroles

## Abstract

In the 1970's, an unconventional stressful photodynamic treatment applied to plants was investigated in two directions. Exogenous photosensitizer treatment underlies direct photodynamic stress while treatment mediating endogenous photosensitizer over-accumulation pinpoints indirect photodynamic stress. For indirect photodynamic treatment, tetrapyrrole biosynthesis pathway was deregulated by 5-aminolevulenic acid or diphenyl ether. Overall, photodynamic stress involves the generation of high amount of reactive oxygen species leading to plant cell death. All these investigations were mainly performed to gain insight into new herbicide development but they were rapidly given up or limited due to the harmfulness of diphenyl ether and the high cost of 5-aminolevulinic acid treatment. Twenty years ago, plant photodynamic stress came back by way of crop transgenesis where for example protoporphyrin oxidases from human or bacteria were overexpressed. Such plants grew without dramatic effects of photodamage suggesting that plants tolerated induced photodynamic stress. In this review, we shed light on the occurrence of plant photodynamic stress and discuss challenging issues in the context of agriculture focusing on direct photodynamic modality. Indeed, we highlighted applications of exogenous PS especially porphyrins on plants, to further develop an emerged antimicrobial photodynamic treatment that could be a new strategy to kill plant pathogens without disturbing plant growth.

## Introduction

**Figure 1:**
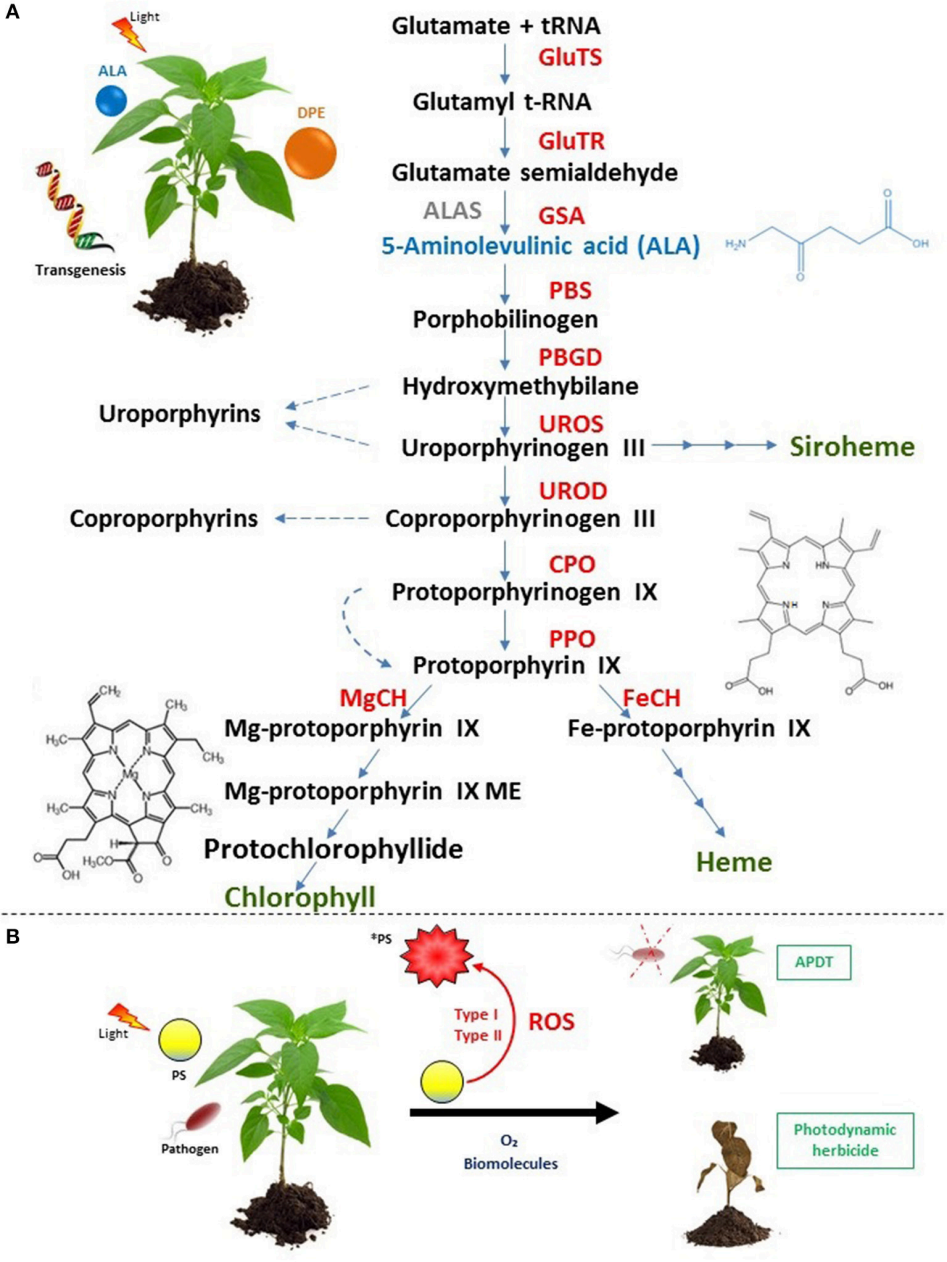
Occurrence of plant photodynamic stress. **(A)** Indirect photodynamic stress occurs through forcing plants to over-accumulate endogenous PS upon tetrapyrrole biosynthetic pathway deregulation by ALA or DPE treatments as well as by transgenesis. Enzymes (capital letters) are shown in red. End products of the pathway are shown in green preceded by multi-arrows. Dashed arrows indicate the generation of endogenous PS (uroporphyrins, coproporphyrins, protoporphyrin IX that can be free-base or Fe/Mg metalated or esterified and protochlorophyllide) with the corresponding structures of the main harmful PS: protoporphyrin IX and protochlorophyllide. The non-plant enzyme ALAS is shown in gray. The first common precursor ALA is shown in blue with its chemical structure. ALAS, 5-aminolevulinic acid synthase; CPO, coproporphyrinogen oxidase; FeCH, Fe-chelatase; GSA, glutamate-1-semialdehyde aminotransferase; GlutTR, glutamyl-tRNA reductase; GluTS, glutamyl t-RNA synthetase; PBGD, porphobilinogen deaminase; MgCH, Mg-chelatase; Mg-protoporphyrin IX ME, Mg-protoporphyrin IX methyl ester; PPO, protoporphyrinogen oxidase; UROD, uroporphyrinogen decarboxylase; UROS, uroporphyrinogen synthase. **(B)** Direct photodynamic stress is carried out via the use of exogenous PS such as porphyrins, phenothiazinium dyes, coumarins, and furocoumarins leading to ROS generation via two types of photochemical reactions upon irradiation. It was recently investigated for applications in agronomy in the context of APDT and for new herbicide development. ^*^PS, excited PS.

Almost all abiotic stresses induce oxidative stress underlying imbalance between reactive oxygen species (ROS) production and plant defense systems (Ramel et al., 2012; Müller-Xing et al., 2014; Hu et al., 2015; Loreti et al., 2016; Vian et al., 2016; Chakradhar et al., 2017; Hasan et al., 2017; Jaleel et al., 2017; Ohama et al., 2017; Rihan et al., 2017; Yang and Guo, 2017). Often, photo-oxidative and photodynamic stresses are confused whereas they bear two distinct meanings. The former points out a light-driven generation of ROS in chloroplasts through the photosensitization of excited chlorophyll molecules that are embedded in antennae complex and reaction center or via electron leakage from overloaded electron transport chain within photosystem apparatus. However, photodynamic stress involves the accumulation of exogenous or endogenous PS at various subcellular compartments and subsequently photochemical ROS production via two types of photochemical reactions under light conditions. In the type I, redox state change of excited sensitizer occurs upon reactions with biological molecules and oxygen resulting in hydrogen peroxide and free radical generation while In the Type II, energy from excited PS is transferred directly to oxygen leading to singlet oxygen production (Figure 1; Foyer et al., 1994; Pospíšil, 2016; Kashef et al., 2017).

In plants, occurrence of photodynamic stress corresponds to two distinct artificial situations. The first one involves deregulations of plant tetrapyrrole biosynthetic pathway using molecules such as 5-aminolevulinic acid (ALA), diphenyl ether (DPE) or genetic tools. Tetrapyrroles play numerous roles from light harvesting, oxygen transport, oxidative phosphorylation, oxygen storage, nitrogen fixation to ROS scavenging (heme) (Figure 1A; Battersby et al., 1980; Senge et al., 2014, 2015). Under normal conditions, this primary biosynthetic pathway is tiny regulated and mainly confined to plastidial organelles that protect cells from potential or accidental oxidative burst. Nevertheless, when this pathway is not anymore regulated by for instance exogenous supply of ALA or DPE or genetic modifications, some intermediates such as protoporphyrin IX (PPIX) and Mg-porphyrins become powerful photosensitizers that could trigger carbohydrates, proteins, lipids and nucleic acids damages (Rebeiz, 2014). In the second situation, photodynamic stress is induced through plant exposure to exogenous PS which are able to produce high amount of ROS inside cells under irradiation. Applications of exogenous PS such as phenothiazinium dyes, coumarins and furocoumarins, porphyrins were performed and summarized in Table 1. A new application of photodynamic treatment on plants is raising up as efficient weapon to struggle against pathogens essentially bacteria and fungi in the context of the so-called antimicrobial photodynamic treatment (APDT) (Figure 1B). Indeed, within APDT, plants of agronomic interest will normally grow protecting themselves from deleterious effect of photoactivated PS via setting up powerful antioxidant machinery. This review will develop photodynamic stress in plants and focus on the direct photodynamic stress regarding APDT to gain insight in improving agronomic practices with high crop yield and environmental protection goals. Photodynamic strategy applied to pathogens or microorganisms is subject of numerous reviews and will not be developed here (Ben Amor and Jori, 2000; Jori and Brown, 2004; Maisch, 2007, 2009; Donnelly et al., 2008; Almeida et al., 2011; Jori, 2011; Alves et al., 2015; Liu et al., 2015; Tim, 2015; Hamblin, 2016; Wainwright et al., 2016; Kashef et al., 2017).

**Table 1:** Exogenous PS and their characteristics and application in plant photodynamic treatment.

	**PS**	**Origin**	**Concentration**	**Light range**	**Light intensity**	**Plant material**	Function^*^	**References**
Non-porphyrin compounds	Procion yellow	Synthetic	4% solution	UV		Elodea leaf mesophyll cells	Intracellular ultrastructure staining	Goodwin, 1976
	Cercosporin	Natural	Variable (0.5–18.7 μM)	Various types of lamps	Variable	Potato, carrot, red beet, tobacco leaf discs, maize roots and NT575 tobacco suspension cells	Phytotoxin	Macri and Vianello, 1979; Daub, 1982a,b; Daub and Briggs, 1983
	Rose bengal	Synthetic	10 mM	White	100 and 350 μmol.m^−2^.s^−1^	Pea leaf discs	Membrane and nucleus staining	Knox and Dodge, 1984
	Hypericin	Natural	100 μM	White	400 μmol.m^−2^.s^−1^	Pea leaf discs	Plant defense	Knox and Dodge, 1985a
	Eosin Y	Synthetic	5 μM and 1 mM	White and green	350 μmol.m^−2^.s^−1^ and 5.26 mW.cm^−2^	Pea leaf discs and onion bulb roots	Protein staining, DNA binding	Knox and Dodge, 1985b; Molero and Hazen, 1988
	Berberine	Natural	Variable (1 nM to 10 μM)	Violet	195 KW.m^−2^	Roots of onion bulbs	Antimicrobial, heparin staining, DNA binding	Molero et al., 1985
	Pyronin Y	Synthetic	1 and 5 μM	Green	129.9 KW.m^−2^	Roots of onion bulbs	DNA binding	Armas-Portela et al., 1985
	Acridin orange	Synthetic	5 μM	Green	5.26 mW.cm^−2^	Roots of onion bulbs	Mitochondria staining and DNA binding	Molero and Hazen, 1988
	Orcein	Natural	5 μM	Green	5.26 mW.cm^−2^	Roots of onion bulbs	Chromosome staining	Molero and Hazen, 1988
	Harmine	Natural	500 nM	UV	2.5 mW.cm^−2^	Roots of onion bulbs	enzymatic inhibition and DNA binding	Hazen and Gutierrez-Gonzalvez, 1988
	Coumarins and furocoumarins	Natural	Variable	Solar radiation		Citrus tree leaves and strawberry leaves	Plant defense	de Menezes et al., 2014a; Fracarolli et al., 2016
	Phenothiazinium dyes	Synthetic	Variable (5, 25, and 50 μM)	Solar radiation		Citrus tree leaves (healthy and combinated with fungal pathogen)	DNA/RNA staining and bacterial staining	de Menezes et al., 2014b; Gonzales et al., 2017
Porphyrins	HPD	Synthetic	25 μg.ml^−1^	Near UV	9 W.m^−2^	*Vicia faba* leaf protoplasts	Usage in PDT as PHOTOFRIN	Kjeldstad et al., 1986
	TPyP and HP	Synthetic	100 nM	Red	0.001 J.m^−2^	Roots of onion bulbs	DNA binding	Hazen et al., 1987
	TMPyP/Zn-TMPyP	Synthetic	Variable (10 nM to 100 μM)	Red, blue, white and solar radiation	Variable	Onion bulb roots, TBY-2 cells, kiwi leaves (healthy and contaminated), tomato and *Arabidopsis thaliana* plantlets	DNA binding	Villaneuva et al., 1986, 1989; Riou et al., 2014; Guillaumot et al., 2016; Issawi et al., 2018; Jesus et al., 2018
	TPPS/Zn-TPPS	Synthetic	3.5 μM	White	95 and 250 μmol.m^−2^.s^−1^	TBY-2 suspension cells, tomato and *Arabidopsis thaliana* plantlets		Riou et al., 2014; Guillaumot et al., 2016; Issawi et al., 2018

*TPyP, Tetra(4-pyridyl) porphyrin; TMPYP, Tetra (N-methylpyridyl) porphyrin; TPPS, meso-tetra (4-sulfophenyl) porphyrin; HPD, Hematoporphyrin derivative; HP, Hematoporphyrin; TBY-2, Tobacco Bright Yellow-2*.

* *Other properties of PS that are not related to their photodynamic action*.

## Indirect photodynamic stress

Forcing plants to accumulate excessive amount of endogenous tetrapyrrolic photosensitizers induce photodynamic stress conditions such as ALA feeding, DPE treatment as well as by transgenesis experiments leading to growth and development impediment. In this review, we will not develop the crop transgenesis tools because they do not fit with plant photodynamic treatment. The reader should refer to theses references for more informations (Li, 2003; Lee et al., 2004; Li and Nicholl, 2005; Jung et al., 2008; Ayliffe et al., 2009; Jung, 2011; Quesada et al., 2013; Yun et al., 2013; Kim et al., 2014).

### 5-aminolevulinic acid (ALA) feeding

5-aminolevulinic acid is not a PS *per se*. Instead, it is a non-protein amino acid and the first common precursor of the tetrapyrrole (chlorophylls, heme, and derivatives) pathway (Figure 1A). Its supply lead to PPIX and/or other intermediates over-accumulation. From the 70's, exogenous application of ALA on yeast, insects, plants and in mammal cells was shown to induce high accumulation of tetrapyrroles (Figure 1A; Brouillet et al., 1975; Rebeiz et al., 1984, 1995; Matsumoto et al., 1994; Juzeniene et al., 2002; Fotinos et al., 2006; Xu et al., 2015). When tetrapyrroles were over-accumulated by ALA feeding, plants could not anymore struggle against induced photodynamic stress and died (Rebeiz et al., 1984, 1990; Matsumoto et al., 1994). When cucumber fields were sprayed with ALA, it was found that seedlings accumulated massive amount of endogenous porphyrins especially the potential singlet oxygen generator “protochlorophyllide” under 5,000 foot candle (Rebeiz et al., 1988). A similar result was obtained on duckweed (*Lemna paucicostata* Hegelm.) that showed rapid membrane damage after light irradiation and increase in both protochlorophyllide and PPIX contents suggesting herbicidal effect of ALA (Matsumoto et al., 1994). In the other hand, ALA-treated plants significantly upregulated transcript levels of genes encoding superoxide dismutase and serine/threonine kinase receptors but the induction of antioxidative components lacked capacity to withstand ROS generation (Phung and Jung, 2014, 2015). In 2004, Jung and co-workers shed light on “photodynamic stress” as they showed that rice plants suffered from severe oxidative damage upon the ectopic expression of the bacterial ALA synthase gene bringing about the accumulation of harmful photosensitizers PPIX and protochlorophyllide under 350 μmol.m^−2^.s^−1^ (Jung et al., 2004). ALA feeding was performed in order to look for a new herbicide. However, there was no commercial formulation of ALA as field effective herbicide owing to the high amount required (≥5 mM) and the cost-effective treatment (Sasikala et al., 1994; Phung and Jung, 2014; Xu et al., 2015; Nguyen et al., 2016).

### Diphenyl ether (DPE) treatment

Since 1960's, DPE essentially oxyfluorfen and acifluorfen were introduced as commercial herbicides to control weeds (Yang et al., 2006). They constitute the main class of PPO-inhibiting herbicides that are widely investigated. Phung and Jung (2015) reported the different responses of photodynamically stressed rice plants undergoing ALA (5 mM) and oxyfluorfen (50 μM) herbicidal treatment. In term of phenotype under illumination, ALA induced bleached necrotic spots while oxyfluorfen caused bronzed necrotic spots on the leaves. This difference in photodynamic symptoms was due to PPIX overaccumulation in cytoplasm in DPE-treated plants whereas the photodynamic destruction of chlorophyll by Mg-porphyrins was responsible of the white spot appearance. Beyond the phenotypical effects, the brown necrosis in DPE-treated plants exhibited a more dispersed H_2_O_2_ production and was accompanied by an increase in H_2_O_2_-scavenging enzymes, catalase and peroxidase activities as well as dehydroascorbate content, a strong stress marker compared to those of ALA-treated plants (Phung and Jung, 2015). Their mode of action was established and consisted in the inhibition of protoporphyrinogen oxidase (PPO) the last enzyme at the branching point between heme and chlorophyll synthesis (Figure 1A; Matringe and Scalla, 1987; Matringe et al., 1989a,b). Thus, triggering leakage of PPO substrate, the non-fluorescent protoporphyrinogen IX that was converted by unknown peroxidase to the first effective PS of this pathway PPIX. Indeed, when PPIX absorbs light, it induces photochemical reactions and vital processes are affected (Figure 1A).

## Direct photodynamic stress: an old story with novel development

Plant exposure to exogenous PS could induce tissue damage and subsequently cell death. First studies relative to direct application of PS on plant materials were reported four decades ago (Table 1). Concerning porphyrins, the most used PS, Kjeldstad and co-workers showed the photodamage of plasma membrane of *Vicia faba* leaf protoplasts subjected to hematoporphyrin derivative treatment under near UV light (Kjeldstad et al., 1986). Moreover, other studies showed mutagenic effect of porphyrins which were able to bind DNA in root meristematic cells of onion bulbs (Table 1; Villaneuva et al., 1986, 1989; Hazen et al., 1987). The aims of testing exogenous PS on plant materials were to study the symplastic intracellular movement, decipher the mode of action of fungal toxin as well as the effects of singlet oxygen on plant cells and exploring sister chromatide exchange upon dye DNA-intercalation in fast-rate dividing cells (Table 1; Goodwin, 1976; Macri and Vianello, 1979; Daub, 1982a,b; Daub and Briggs, 1983; Knox and Dodge, 1984, 1985a,b; Armas-Portela et al., 1985; Molero et al., 1985; Kjeldstad et al., 1986; Villaneuva et al., 1986, 1989; Hazen et al., 1987; Hazen and Gutierrez-Gonzalvez, 1988; Molero and Hazen, 1988).

For agronomic issues, the use of exogenous PS as powerful photoactivated molecules was not anymore investigated because the undesirable effects described above. Hence, any potential application of whatever PS in the aim to fight plant pathogens requires a risk assessment on plant hosts. It was reported that natural photosensitizers such as coumarins and furocoumarins or synthetic ones such as phenothiazinium and porphyrins inactivated pathogenic agents as virus (Tobacco mosaic virus), bacteria (*Pseudomonas syringae*) and fungi (*Collectotrchum abscissum, Colletotrichum gloeosporioides, Collectotrichum acutatum, Aspergillus nidulans, Fusarium oxysporum, Fusarium moniliforme, Fusarium solani*) (Table 1). However, when spotted on orange tree and strawberry plants, or on kiwi contaminated leaves under solar radiation, the leaves and flowers were not affected by either natural/synthetic photosensitizers excepted for strawberry leaves that were damaged upon treatment with 100 μM phenothiazinium (Orlob, 1967; de Menezes et al., 2014a,b; Fracarolli et al., 2016; Gonzales et al., 2017; Jesus et al., 2018). In another extended context, Issawi and co-workers conceived a double target strategy that could eradicate in the same time unwanted vegetation and plant pathogens without killing plants of agronomic interest (Figure 1B). To fulfill that purpose, they studied the effect of exogenous water-soluble cationic and anionic porphyrins on tomato, plant of agronomic interest and on *Arabidopsis thaliana*, weed-like plant. Thus, they showed that cationic porphyrins were able to eradicate Arabidopsis plantlets without killing tomato plantlets (Guillaumot et al., 2016; Issawi et al., 2018). Favorably, Riou and co-workers treated TBY-2 cells with these same porphyrins aiming to find out new herbicides because no new herbicide modes of action were discovered since the last 3 decades (Duke, 2012; Heap, 2014; Riou et al., 2014).

## Determinants of plant photodynamic stress

Plants exposed to various stressors respond by involving mechanisms of sensing and signaling (Tuteja and Sopory, 2008; Mittler and Blumwald, 2010; Suzuki et al., 2012; Pandey et al., 2016; Zhu, 2016; Mittler, 2017). Although plant stress signaling pathways were abundantly investigated, stress sensors remain largely unknown so it is much difficult to detect sensing systems in plants subjected to direct photodynamic stress under unconventional conditions. However, Phung and collaborators outlined the switch photodynamic/drought-tolerance in PPO-transgenic rice under drought conditions explaining how drought determinants reduced porphyrin level in order to elaborate tolerance response through gene expression modulation upon sensing change in tetrapyrrole amount (Phung et al., 2011). Nonetheless seeking for photodynamic sensors represents serious challenge. Exogenous PS exert photodynamic function through the production of ROS including singlet oxygen and hydrogen peroxide which are well known signaling molecules, it is worthwhile to distinguish between primary sensing that may be assigned to the PS *per se* and secondary sensing ascribed to ROS (Laloi et al., 2007; Niu and Liao, 2016; Wang et al., 2016). ABC transporters and TSPO receptor could play role in exogenous PS sensing since they were identified as endogenous tetrapyrrolic receptors (Theodoulou, 2000; Guillaumot et al., 2009). Interestingly, PPIX interacted with Toll-Like Receptor 4 (TLR-4) in mammals. Possible interaction with a putative TLR should be investigated in plant cells (Figueiredo et al., 2007; Tangudu and Spasic, 2017). In term of signaling, exogenous photodynamic action is likely to generate various secondary messengers holding signaling potential like ROS, modified proteins, lipid peroxidation by-products. In addition, photodynamic signaling may also involve cross-talk with phytohormones as abscissic acid, jasmonic acid, salicylic acid, calcium, protein kinases, transcription factors (Tuteja and Sopory, 2008; Suzuki et al., 2012; Zhu, 2016). Thus, studies involving transcription profiling, proteomic and metabolomic approaches should be envisaged upon photodynamic administration of exogenous PS in plants.

## Challenges and perspectives

In the present review, two ways to carry out photodoynamic action on plants were emphasized (i) indirect photodynamic reaction via ALA or DPE treatments and transgenesis, (ii) direct photodynamic reaction through the use of exogenous PS. Comparing to conventional weed management methods using photodynamic DPE herbicides that are commercially available, it was reported that this kind of herbicides were not only toxic to weeds but wildlife was also affected as DPE herbicides were toxic to nestling birds and fresh water polyp, also, they could be toxic to humans. In addition, DPE alone were not expected to control weeds and need the combinational use of chemicals or mulch. Indeed, there are several reported weeds that developed DPE-resistance (Hoffman et al., 1991; Rio et al., 1997; Li and Nicholl, 2005; Beckie and Tardif, 2012; Reed et al., 2018). Thus, we conclude that direct photodynamic treatment holding herbicidal potential via exogenous PS could be a promising approach relying on the fact that weeds cannot induce resistance against PS because they exert a multi-targeted photodynamic action damaging cellular DNA, lipids, carbohydrates and proteins. Taken together, we support the application of exogenous PS as weed control alternative and further studies are required as far as we know that studies concerning that task are very few (Riou et al., 2014; Issawi et al., 2018).

In regard to antimicrobial strategies, APDT was recently envisaged as promising approach to strike against plant pathogens without side effects on plant hosts and environment. In this context, we think that several aspects must be taken into consideration such as whole study of a defined pathosystem should be considered including biological life cycle of pathogen, growth and reproduction phenology of plants and timing of PS application. Moreover, PS administration methods as inclusion in soil aqueous phase, spotting on leaves and field spraying should not be neglected although spraying is the most convenient and reasonable.

## Conclusion

In conclusion, plant photodynamic stress is considered as abiotic stress linked to ROS production as the first cause of cell death. It is still poorly studied regarding characterization of photodynamic stress determinants and outcomes especially at molecular level in plants. Besides, plant photodynamic stress has not been exploited yet, especially as valuable exogenous treatment for the purposes we mentioned above. Nevertheless, we are confident that in the near future, this approach based on PS and especially porphyrins could be relevant to respond to the Directive 2009/128/EC of the European Parliament plans that aim to reduce the use of pesticides while maintaining high yield as well as high quality in agricultural production. PS are photodegradable and non-toxic under dark as well as they were used at micromolar concentrations therefore they could be promising candidates to fulfill the task of European projects for environment sustainability in respect to wildlife, water sources and human health.

## Author contributions

MI and CR prepared and wrote the present manuscript. MI designed the figure and the table. VS reviewed the manuscript.

### Conflict of interest statement

The authors declare that the research was conducted in the absence of any commercial or financial relationships that could be construed as a potential conflict of interest.
